# Improving preservation state assessment of carbonate microfossils in paleontological research using label-free stimulated Raman imaging

**DOI:** 10.1371/journal.pone.0199695

**Published:** 2018-07-11

**Authors:** Asefeh Golreihan, Christian Steuwe, Lineke Woelders, Arne Deprez, Yasuhiko Fujita, Johan Vellekoop, Rudy Swennen, Maarten B. J. Roeffaers

**Affiliations:** 1 Department of Earth & Environmental Sciences, Division of Geology, KU Leuven, Heverlee, Belgium; 2 Department of Chemistry, Centre for Surface Chemistry and Catalysis, KU Leuven, Heverlee, Belgium; 3 Department of Chemistry, Molecular Imaging and Photonics, KU Leuven, Heverlee, Belgium; Institute of Materials Science, GERMANY

## Abstract

In micropaleontological and paleoclimatological studies based on microfossil morphology and geochemistry, assessing the preservation state of fossils is of the highest importance, as diagenetic alteration invalidates textural features and compromises the correct interpretation of stable isotope and trace elemental analysis. In this paper, we present a novel non-invasive and label-free tomographic approach to reconstruct the three-dimensional architecture of microfossils with submicron resolution based on stimulated Raman scattering (SRS). Furthermore, this technique allows deciphering the three-dimensional (3D) distribution of the minerals within these fossils in a chemically sensitive manner. Our method, therefore, allows to identify microfossils, to chemically map their internal structure and eventually to determine their preservation state. We demonstrate the effectiveness of this method by analyzing several benthic and planktonic foraminifera, obtaining full 3D distributions of carbonate, iron oxide and porosity for each specimen. Subsequently, the preservation state of each microfossil can be assessed using these 3D compositional maps. The technique is highly sensitive, non-destructive, time-efficient and avoids the need for sample pretreatment. Therefore, its predestined application is the final check of the state of microfossils before applying subsequent geochemical analyses.

## Introduction

Microfossils provide a powerful tool in paleoecological, paleoceanographical, paleoenvironmental and paleoclimatological research. Species distributions, morphological variations and test (shell) geochemistry are commonly used to reconstruct paleoenvironmental conditions such as substrate, water depth, and seawater chemistry [1]. As microfossil tests have the capability to preserve trace elemental and stable isotope signals, they enable the reconstruction of ambient sea water chemistry and temperature. Consequently, the identification of microfossils based on their unique visual features and determination of their stable isotopic and trace elemental signature are primary and daily practices in paleoclimatological and micropaleontological studies. Pivotal to these practices is the improvement of analytical techniques and protocols to accurately identify each specimen or species and to subsequently select eligible samples for trace elemental and stable carbon, oxygen and clumped isotopic investigations [2].

A first paleotemperature study using the oxygen isotopes of carbonate fossils was published by Urey *et al*. (1951). Since then, there have been countless follow-up studies, as stable isotopic analyses have become standard practice in paleoclimatological research [3]. However, several misinterpretations have been reported in literature, originating from artifacts like recrystallization and contamination by cement, all in relation to the diagenetic alteration of the microfossil carbonate [4]. For instance, “the cool tropic paradox” reported by D’Hondt and Arthur (1996) during the Cretaceous and the Eocene [5], was likely caused by diagenesis [6, 7]. Diagenetic alteration (classification further below) provokes problems in accurate paleotemperature reconstruction by compromising the pristine isotopic signature [8–12]. Yet, so far, no direct chemical proxy or monitoring technique exists to indisputably track altered oxygen isotope ratios upon diagenetic influences [13]. Therefore, studying the preservation state and the detection of diagenetic alteration of fossils is critical before doing any stable isotopic or trace elemental analysis for paleotemperature reconstructions.

**Fig 1:**
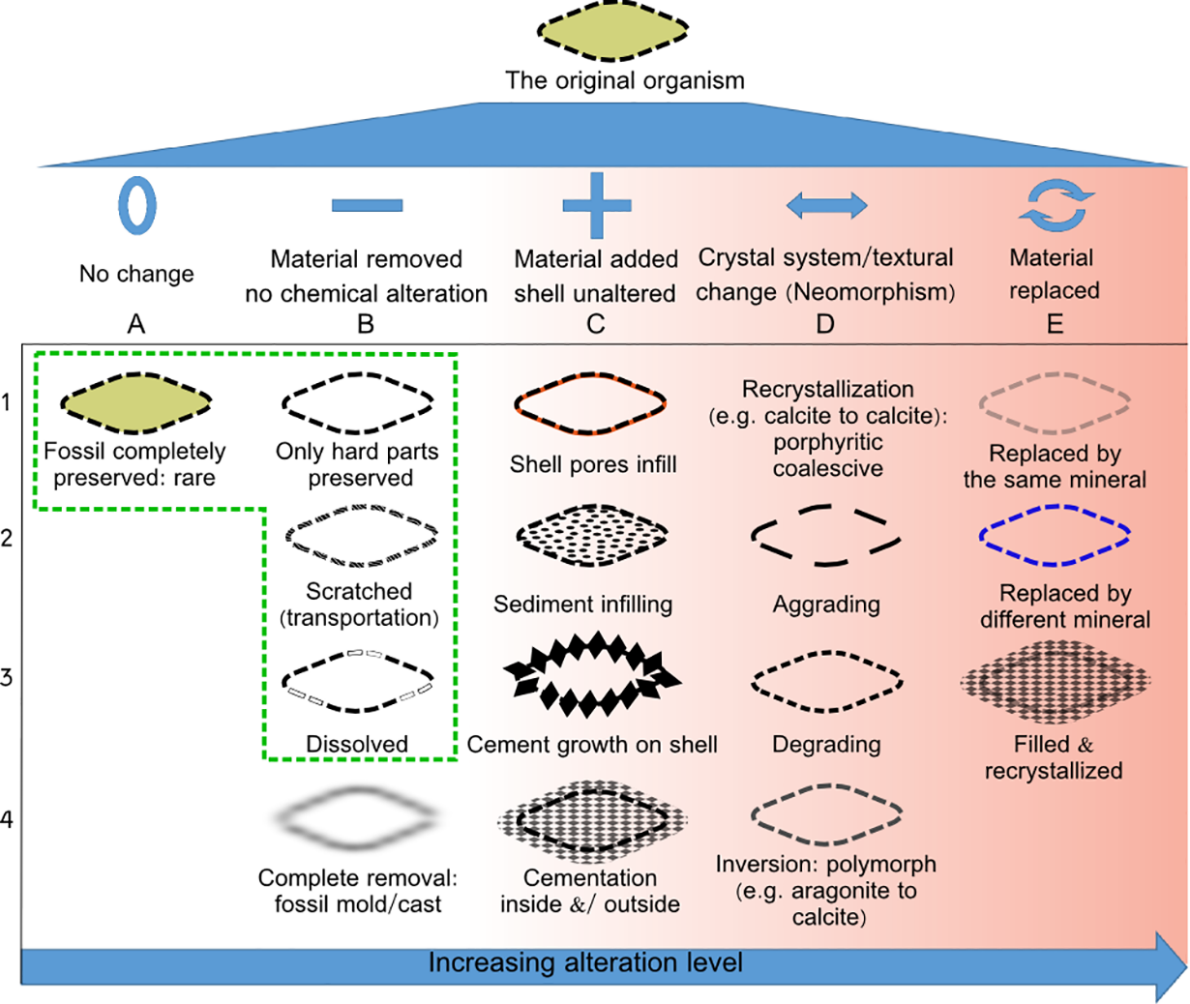
Schematic illustration of preservation state of a microfossil in different conditions. Fossils can be fully preserved, including soft tissues (A1), however, this is rare and mainly happens upon immediate burial. Most of the time, alteration happens to some extent which can include the removal of material (B), inclusion of other material (C), change of the crystal system by present material (D) or replacement of material by other matter which can possess the same or different mineralogy (E). In detail: (B1) Soft parts of the fossil are removed and hard parts remain without change. (B2) Scratches on the fossil test can occur due to transportation. This corresponds to physical removal of material from the shell’s surface. (B3) Chemical dissolution of the fossil due to a change in diagenetic environment. (B4) Fossil molds: the fossil is completely removed but a mold or cast is preserved. (C1) The test pores are filled by secondary minerals, with the same or a different mineralogy. (C2) Sediment infilling which is normally soft and can be washed away, e.g. by ultrasonic treatment. (C3) Crystal overgrowth on the test. (C4) Cementation inside and/or outside fossil. (D1–3) Neomorphism (recrystallization) by changing the crystal size or texture with preservation of same mineralogy. (D4) Neomorphism (inversion) by changing the crystal system or polymorph. (E1) Replacement of the primary mineral by a secondary mineral which basically has the same mineralogy but has a different isotopic signature. (E2) Replacement of the fossil test by a different mineral. (E3) Combination of adding material and alteration of primary test. The area marked with a green dashed line shows states valid for accurate isotope measurements. Alteration levels increase from (A) to (E).

The fossil’s post-depositional degree of alteration is strongly related to the susceptibility of the system to changes. This is varying from open or water dominant to closed or rock dominant alteration [14]. Not all preservation changes are affecting the isotopic signature in the same way, as there are diversified modes of alterattion. The preservation state is normally divided into several categories: a) preservation of pristine fabric, which is rare; b) preservation of the hard parts, which are either unaltered or reflect different levels of alteration; c) complete removal of fossil and preservation of external mold or cast. A schematic illustration of different preservation states is presented in Fig 1. The processes describing alteration include permineralization, neomorphism (inversion, recrystallization), replacement and cementation [15, 16].

Permineralization describes fossilization by mineral formation inside the pores or alternatively sediment infill. To recognize permineralization, information from the inside of a sample is required. This can be achieved by invasive methods such as sample fractioning, by non-invasive computed tomography [17] or simply by investigating transparency changes [4]. Neomorphism is the process of a mineral changing to a different polymorph or texture *i*.*e*. inversion (e.g. aragonite to calcite) or recrystallization (e.g. calcite to calcite with a different crystal habitus) [15].

Growth of secondary crystals on the shell or inside of a fossil which changes the original fine structure is classified as cementation. These can be identified by a mineralogical and morphological assessment of the sample.

Finally, crystal replacement happens when the original mineral dissolves and is superseded by a different mineral (e.g. silica, pyrite, hematite, or calcite infill in the fossil molds) which can even happen atom by atom. This can potentially be recognized by mineralogical analysis but if the exchanged mineral possesses the same mineralogy as the original fossil, it may be hardly noticeable.

Considering all changes that can affect microfossils, a detailed investigation of morphology and composition is vital before performing any kind of isotopic analysis. In sedimentary records where investigated microfossils are relatively rare, often only a few specimens per sample are available for geochemical analyses. Therefore, assessments of the preservation state of individual carbonate microfossils are preferentially non-destructive, not affecting the purity of samples, and not modifying their geochemical signature, so the tested specimens can directly be used for geochemical analyses.

Many experimental methods have been used in paleontological research to assess the preservation of microfossils based on surface morphology, internal structure, and geochemistry. Those mainly include optical microscopy (stereo, transmitted light, and confocal fluorescence microscopy), Raman microspectroscopy, micro-computed tomography (μ-CT), environmental, scanning or transmitted electron microscopy (ESEM, SEM, TEM), secondary ion mass spectrometry (SIMS, time-of-flight TOF-SIMS, and NanoSIMS), synchrotron tomography, laser ablation ICP-MS, and/or a combination of several techniques [11, 18–21]. Combined techniques can also be used to perform both 3D visualization and 3D quantitative analysis. Such techniques include focused ion beam milling combined with electron microscopy (FIB-SEM or FIB-TEM, respectively). Also destructive geochemical analysis, such as Sr-analysis, were used to assess potential diagenetic alteration [22].

The preservation state, in particular, is often investigated by binocular microscopy and SEM [4, 23, 24]. According to Sexton *et al*. (2006), there are three categories describing the preservation state of microfossils based on transparency changes of fossils during studies in dry and wet state by stereo microscopy: glassy, frosty and chalky [4]. Although binocular microscopy can provide a crude estimation of the general preservation state of the specimens, only higher resolution SEM images can subsequently confirm the frosty nature of foraminifera samples, for example, exhibiting invisible micron-scale cemented overgrowths. However, typical electron microscopy procedures involve the modification, contamination, or even destruction of the sample since coatings (e.g. sputtering) or glue are used in SEM or ESEM, rendering it useless for further isotopic analysis. Furthermore, both techniques fail to acquire a detailed 3D tomography and mineralogy of the entire studied specimens. For instance, a microfossil could show no or few signs of chemical alteration on the outside, while it could be cemented on the inside. Computed tomography is the only non-destructive three-dimensional technique that has been described so far, revealing information about overall (internal and external) morphology. However, special stabilization procedures in μ-CT, visualization, artifacts minimization and the 3D reconstruction themselves are time-consuming. Furthermore, inferring mineralogy of for example microporous samples from tomodensity is not straightforward and it is challenging for minerals with a similar absorption index.

**Fig 2:**
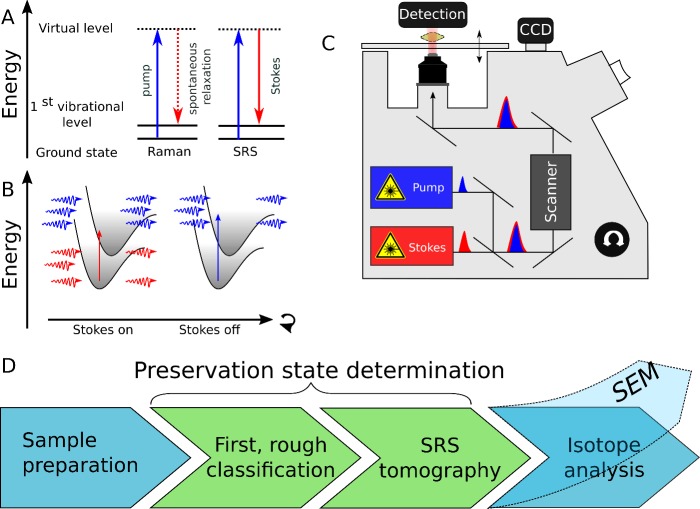
Principle of SRS microscopy (in paleoclimate research). (A) In spontaneous Raman scattering, the Pump photon (in blue) is scattered spontaneously and inelastically, and gets converted into a red-shifted Stokes photon. In SRS, Stokes photons are irradiated to stimulate the Raman transition of interest. The difference between Stokes and pump wavelength corresponds to the energy difference of the targeted vibrational levels. Dashed lines indicate virtual, short-lived molecular levels, solid lines denote real vibrational ones. Sensitive detection of the signal intensity relies on the rapid ON-OFF switching of the Stokes beam. When the Stokes beam is ON, Raman transitions will be stimulated leading to a decrease in pump laser intensity after the sample. When switching the Stokes beam OFF the Raman transition is no longer stimulated and the pump laser will again increase in intensity. Rapid modulation (10 MHz) of the Stokes laser leads to a modulated pump beam transmission which is detected and translated into a signal which is a quantitative measure of the number of Raman scatterers at that frequency (S1 Fig). (B) Pump-probe spectroscopy, based on transient absorption bleaching or ground state depletion, can be used to identify minerals with a strong light absorption i.e. colored such as iron oxides. Here a similar approach as in A is followed however now both lasers are in resonance with the electronic transition e.g. iron oxides etc. When the Stokes beam is ON, they will be absorbed by the colored species leading to a temporary depletion of the ground state. As a result, the pump photons of the second laser beam will no longer be absorbed. Switching the Stokes beam OFF results in the more efficient absorption of pump photons. Detection of these changes in pump beam intensity, based on the rapid modulation (10 MHz) of the Stokes laser, can be translated into a signal which is a quantitative measure of the presence of colored species at that location. As absorption bands (electronic resonances) are spectrally much broader than the corresponding vibrational resonances these can easily be discriminated from each other by hyperspectral SRS imaging. Note that the nomenclature of pump-probe is misleading in this context and adapted to our particular experiment which is primarily used for SRS where the Stokes laser is modulated. In a classical pump-probe experiment, the Stokes beam would be called the pump source. (C) In an actual experiment the tunable pump and Stokes lasers are tuned into a vibrational (SRS) or electronic (pump-probe) resonance of interest and coupled into a microscope for focusing onto the sample. Raster scanning the focus spot over the sample yields 2D (XY) images. Additional movement up and down of the objective with respect to the sample enables obtaining consecutive Z-sections. The detection module (more information in the supplementary information) is connected to a computer where tomographies are reconstructed. Additionally, a CCD camera in combination with a normal white-light source is used to record optical transmission images. (D) Flowchart of a paleoclimate data collection. After excavation and processing (e.g. sieving) of a sample, an initial pre-screening is performed. Microfossils found potentially interesting are thoroughly investigated using SRS chemical and spatial tomography. Samples classified as well preserved are being transferred to isotope analysis. To showcase that SRS is a well-suited technique in paleoclimate analysis we confirm the preservation state using SEM in this paper (dashed lines) after SRS measurements were done—which is not necessary.

Raman spectroscopy allows detecting diagenetic alteration of microfossils in a non-destructive way using photons to probe polarizable bonds by inelastic scattering. Raman scattering is advantageous as the vibrational resonance of specific chemical bonds can be probed simply by irradiating with monochromatic laser light (Fig 2A). Information about the type and number of molecular bonds in a sample then delivers the chemical composition and thus provides a fingerprint of the sampled material. As an established technique that provides information on the original (bio)chemistry of the organism [25], Raman spectroscopy belongs to the toolbox of geochemical research. Luckily, many minerals are Raman active, which is exploited by several studies for solving paleontological problems. For example, Raman spectroscopy has been used to study carbonaceous matter in highly metamorphosed rocks [26], microfossils preserved in chert [27], and for 3D mapping of microfossils [28]. Furthermore, Raman spectroscopy is a well-established technique for polymorphs characterization [29, 30]. This is relevant for recognition of neomorphism of primary fossils (Fig 1 D4). Additional studies using Raman spectroscopy, however, are hindered by the fact that it is inevitably insensitive and hence slow: only a small fraction of incident photons get scattered inelastically and thus integration times are far too long for efficient chemical mapping and tomography.

Stimulated Raman spectroscopy (SRS) offers a solution to the lack of imaging speed typically encountered in spontaneous Raman scattering. In SRS, the weak Raman scattering process is enhanced by externally stimulating the spontaneous Raman transition using a second laser beam. This ‘Stokes’ laser beam must be in resonance with the Raman transition of interest (see Fig 2A) [31, 32]. Effectively, photons of the pump laser beam get converted into Stokes photons and this intensity difference is measured with high precision and translated into chemical mapping information. Due to stimulation of specific Raman transitions, SRS enables data acquisition and imaging orders of magnitudes faster than spontaneous Raman scattering and hence ideally suited for optical label-free chemical tomography of samples e.g. in biomedical research and to a lesser extent in materials research [33].

Also, light absorbing species e.g. iron oxides or pyrite, not displaying a sharp Raman resonance in the probed spectral region, can be detected with this approach. As the electronic states, responsible for the light absorption, are energetically much broader than the discrete vibrational levels, the spectral response of this species, commonly explored in pump-probe imaging, is much broader than the SRS signal. This distinct difference in behavior allows to discriminate both processes. This modality can be exploited alongside SRS as the detection mechanism is similar to generate more detailed information of the sample under study (see Fig 2B) [31, 34].

In this work, SRS is used to generate an accurate reconstruction of the microfossils based on its composition in three dimensions. We show that SRS microtomography in combination with pump-probe spectroscopy is a useful tool for determination of sample preservation in the typical workflow of paleontological research in general and more specifically in paleoclimatological research. The untouched sample can directly be used for further isotope analysis and other measurements. The method can be exploited to identify microfossil species, verifying their preservation state as well as their mineralogical heterogeneity with regards to cementation and other diagenetic alteration.

## Materials and methods

Detailed information on the materials and methods can be found in the supplementary information. This includes a description of all sample characterization techniques and further sample parameters.

**Fig 3:**
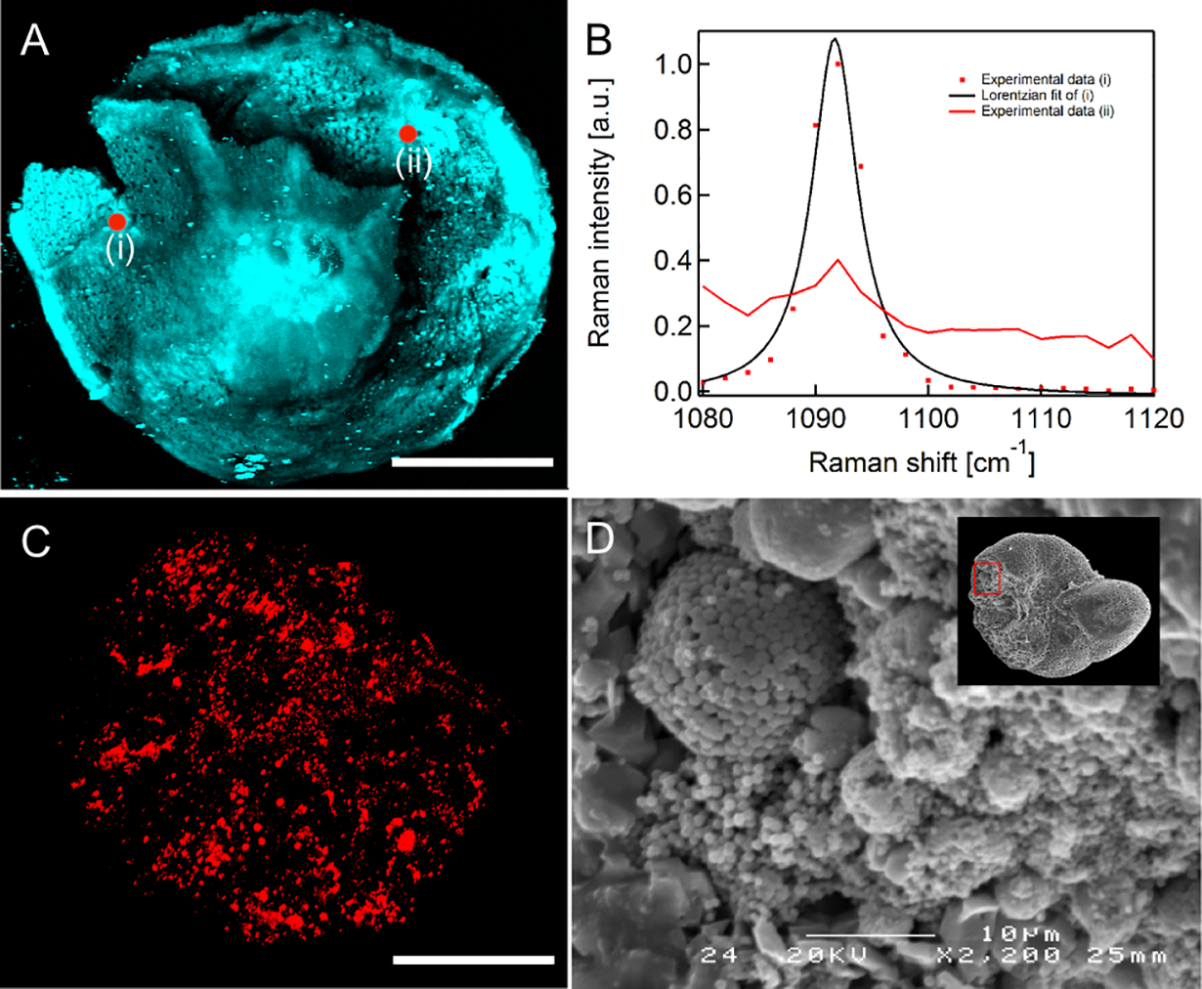
Compositional tomography of benthic foraminifera microfossils using stimulated Raman microspectroscopy. (A) SRS imaging of *Nuttallides truempyi* at 1092 cm^-1^. Shown is a maximum projection of a tomography stack of the sample. (B) The SRS spectrum taken at the indicated position (i) in *Nuttallides truempyi* confirms that Mg calcite is abundant in this sample (data in red, Lorentzian peak fit in black). Tuning the laser off resonance reveals broadly resonant features inside the fossil. Shown here is a scan at an indicated position (ii). (C) An ‘off resonance’ tomography (maximum projection) of *Anomalinoides midwayensis* is displayed with many absorptive features. (D) Magnified SEM image of the same fossil showing framboidal pyritizations inside the test wall. Scale bars of A and C are 80μm.

We propose to follow an experimental scheme for acquiring geochemical data from carbonate microfossils, using SRS tomography as illustrated in Fig 2D. After microfossil excavation and identification, fossils are selected either with stereo microscopy or transmitted light microscopy, then SRS tomography is performed to assess the preservation state. Afterwards, we employ SEM imaging to further support the SRS results (Fig 3–Fig 6).

**Fig 4:**
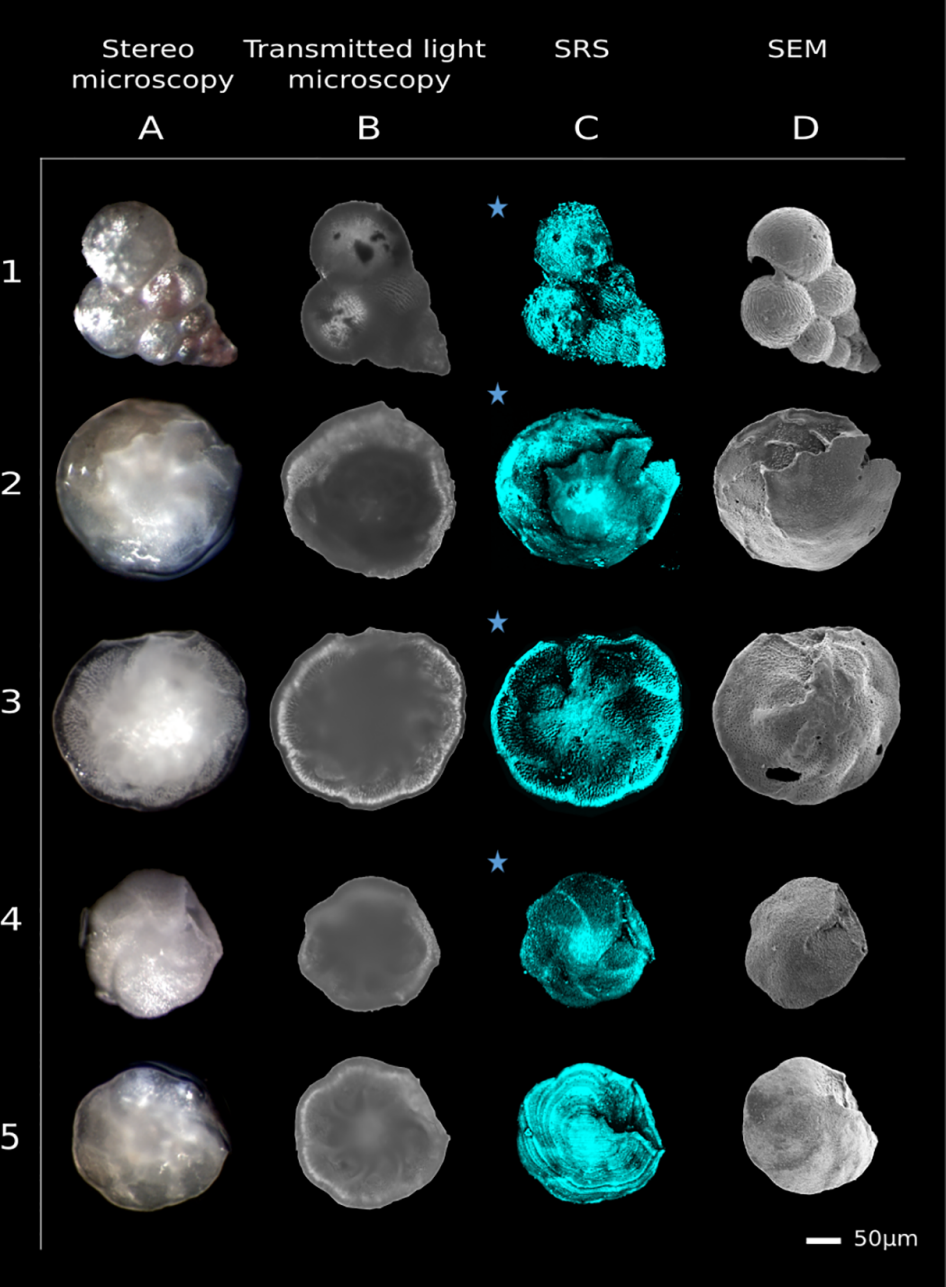
SRS tomography for architecture assessments. Comparison between stereo (A), transmitted light (B), maximum projected SRS (at 1092 cm^-1^) (C) and SEM images (D). Specimen 1 is a planktonic foraminifer of the *Heterohelix* genus with some infillings. Fossils 2 to 5 represent the benthic foraminiferal species *Nuttallides truempyi*. Severely diagenetically altered foraminifera are marked by a star.

**Fig 5:**
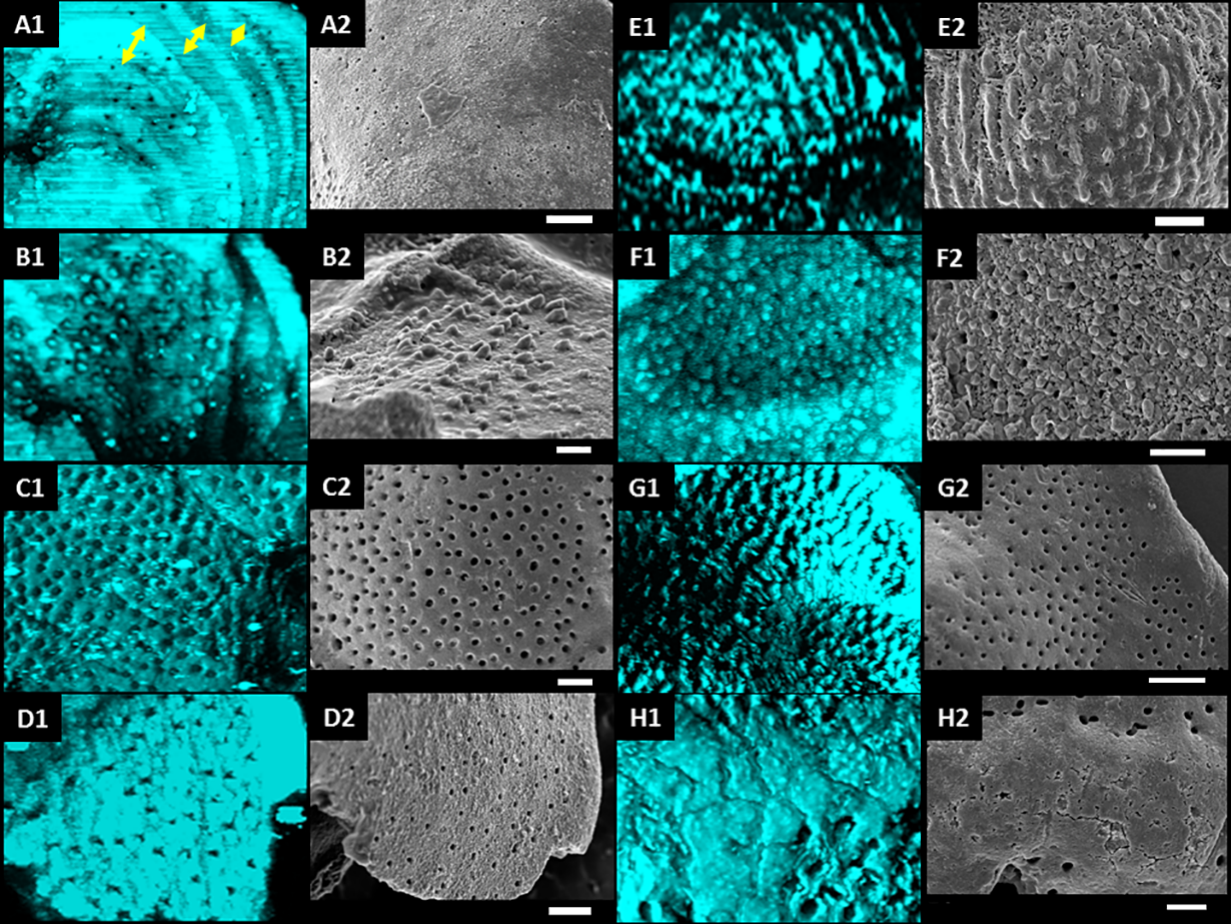
Details of the walls of samples imaged with SRS (maximum projection, visualizing shell and also internal structures) (1) and SEM (2). (A) *Nuttallides truempyi* (Fig 4 row 5) with growth rings visible in A1 indicated by yellow arrows. (B) Detail of Fig 4 row 5 with spines close to aperture (C) *Anomalinoides midwayensis* with some iron oxide coating on surface. (D) Not well preserved *Nuttallides truempyi* (Fig 4 row 2). (E) *Heterohelix* test with oriented pustules (Fig 4 row 1). (F) *Nuttallides truempyi* from Fig 4 row 4 showing obstructed pores and high degree of calcite growth. (G) *Nuttallides truempyi* (Fig 4 row 3) with crystal overgrowth on inner wall test (G1) while the outer part (G2) is nicely preserved. (H) Dissolution marks along microfracture on outer shell part.

**Fig 6:**
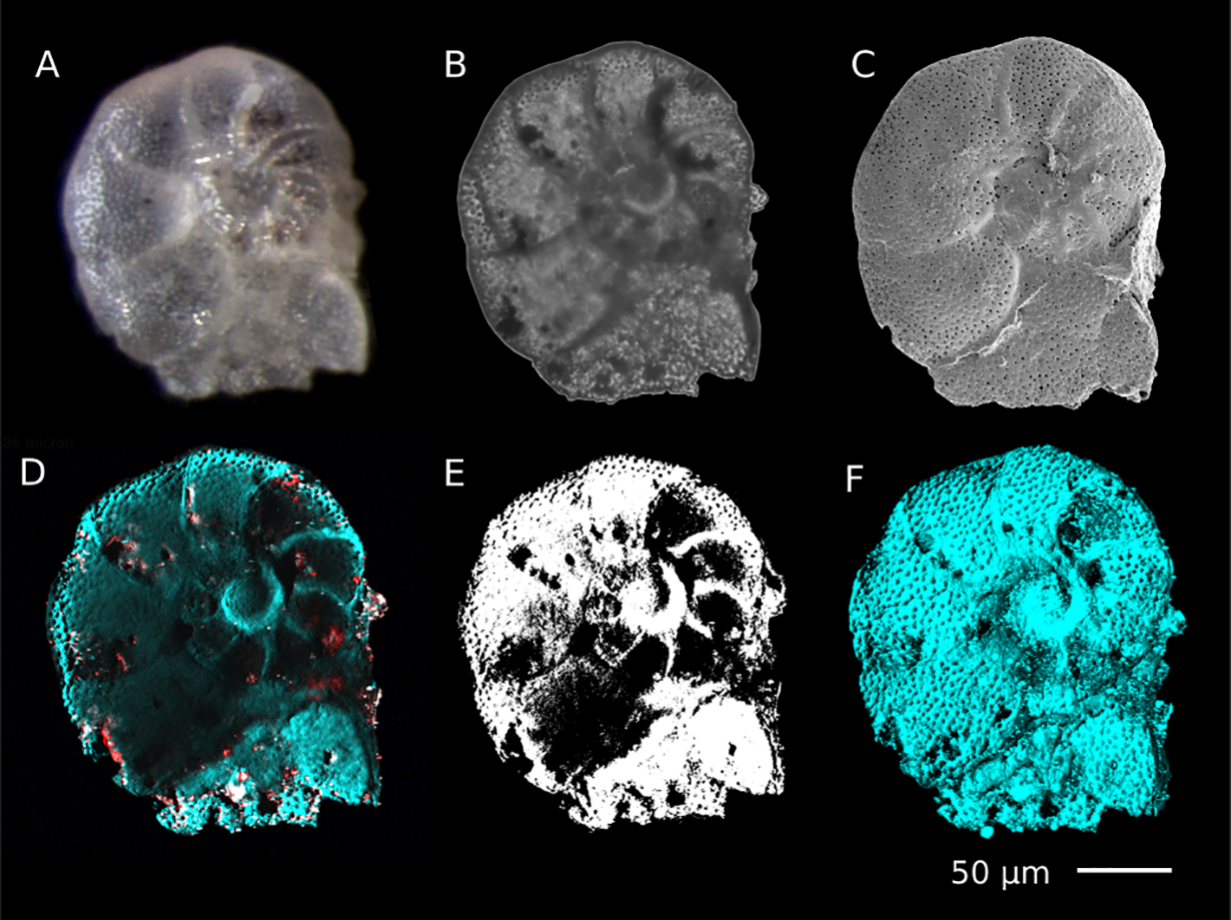
Illustration of advantages offered by SRS micro-imaging for preservation state assessment and volumetric calculations over other typically applied techniques. (A) Stereo microscopy image, (B) transmitted optical microscopy and (C) SEM image of *Anomalinoides midwayensis*. (D) Compositional image taken 35 μm deep inside the microfossil; turquoise color represents Mg calcite at 1092 cm^-1^ and red displays the distribution of iron oxides and pyrite at the same depth by probing absorptive species ‘off resonance’. (E) Segmented image calculated from (D) (white is minerals, black is porosity) showing the porosity distribution 35 μm below the shell surface. (F) Maximum projection of the whole image stack containing a number of SRS images spaced each 0.5 um in depth. (See also Supporting Information for a movie showing the full 3D reconstruction).

We selected a set of fossil foraminifera from four different locations and three different geological ages (see supplementary information for more details on the ages and origin of the studied specimens). Fossilized benthic foraminifera of the species *Anomalinoides midwayensis* (Fig 3C and 3D and Fig 6) and *Nuttallides truempyi* (Fig 3A and Fig 4, rows 1–4), and fossilized planktonic foraminifera of the *Heterohelix* genus (Fig 4, row 5) are scrutinized. This set included both well-preserved and poorly preserved specimens, to assess the full potential of SRS tomography.

Firstly, 10 benthic foraminiferal tests were imaged by stereo microscopy from different sides in dry and wet state to identify the degree of recrystallization by transparency, as can be seen in Fig 4, column A and Fig 6A. At the same time, transmitted light microscopy images were collected (see Fig 4, column B and Fig 6B).

Secondly, SRS data from these foraminifera were collected to reconstruct their architecture and taphonomy in more detail. After determining the test material to be Mg calcite, using spontaneous Raman imaging (see S2 Fig), SRS imaging at the Mg calcite resonance of 1092 cm^-1^ was done on tests immersed in water as required by the used objective. Three-dimensional SRS stacks were taken by z-sectioning the sample with 0.5 micrometer axial stepping distance and the stacks maximum projected in Fig 3A and 3C, Fig 4, column C, Fig 5 column 1 and Fig 6A–6C. Average powers of 30 mW in each beam were applied. A typical setup for SRS tomography with the most vital components is presented in Fig 2C. For more and detailed information on the employed setup see the supplementary materials.

After all non-invasive measurements were done and the samples were no longer needed for optical microscopy, they were sputter-coated with Au-Pd (3 nm thickness) for improved SEM analyses. SEM experiments were performed *after* all non-invasive measurements because of the applied Au-Pd coating which would interfere with all other techniques. Subsequently, SEM images were acquired (Fig 4, column D, Fig 5 column 2 and Fig 6C). In the last step, we fractioned and recoated the samples and acquired SEM images from the freshly broken surface to confirm infillings and cementation inside the test wall (Fig 3D).

## Results and discussion

### Compositional tomography of microfossils via stimulated Raman microspectroscopy

It is clear that an important advantage offered by SRS microtomography over existing approaches is the option to perform detailed chemical mapping of a whole microfossil. To demonstrate the capability of SRS as a sensitive tool to assist in the assessment of the preservation state of microfossils and specifically volumetric calculations, the fossil benthic foraminifera *Anomalinoides midwayensis* was scanned using SRS ‘on resonance’ of the symmetric CO_3_ stretching of Mg calcite at 1092 cm^-1^, as well as ‘off resonance’ [35] (Fig 3A).

The SRS spectrum (Fig 3B) shows the dominant spectral feature of the sample at 1092 cm^-1^ (FWHM = 5 cm^-1^), which is characteristic for Mg calcite and in agreement with spontaneous Raman spectroscopy (see S2 Fig). Indeed, this mineral is the building material of the foraminifera under study. The 3D distribution of other (absorbing) minerals was mapped out by hyperspectral imaging (1080–1120 cm^-1^) enabling to discriminate between the sharp vibrational resonance of CO_3_ and the broad spectral response of colored species and mineral. A comparison in Fig 3B clearly shows this difference between vibrational and electronic resonances. Light absorbing species with a broad spectral response are mapped out specifically in red in a different fossil in Fig 3C. Most likely these light absorbing species in this specific sample are iron oxides and pyrite. The SEM image in Fig 3D confirms this assumption as framboidal pyrite is clearly present in these fossils. As iron oxides and pyrite are not primary minerals, they constitute a form of contamination such as shell coating or infilling, which would also complicate trace elemental analyses on this specimen. SRS mediated tomography of the sample delivers valuable information on the distribution of main and spurious minerals inside a foraminifer.

### Comparison of SRS microscopy imaging of microfossils with established techniques

Stereo microscopy, which is typically the first method used for sample identification and sample selection, does not provide much information on internal structural details (see Fig 4, column A, brighter spots are due to reflections). Furthermore, from stereo images details of the shell surface are not clearly visible (Fig 4, compare column A with the rest). While transmitted light microscopy shows more detail than binocular images, internal and fine surface structure information is still missing (Fig 4, column B).

SRS yields higher contrast and better resolved images that reveals the openings (aperture) of the organism and minute details of its shell that were otherwise only visible in SEM (Fig 4, column C and D vs Fig 4, column A and B). This unique characteristic of the technique will be further exploited in the following paragraphs using different microfossils.

### Correct assessment of microfossils preservation state based on shell architecture

The microfossil’s surface morphology is a good indicator of the integrity of the specimen [24]. The **primary structure study** of microfossils (Fig 1A and B1) considers unique features of each specimen or species including shape, size, coiling of the shell (right or left-coiling), aperture shape and position, internal structures, chamber numbers, shape and arrangements and shell surface primary morphology. This facilitates the identification and classification of the fossil. Furthermore studying primary surface structure yields information about environmental conditions during organism life including substrate, water depth, and seawater chemistry [1, 25].

During **secondary structure analysis,** the shell surface modified morphology or shell infill by permineralization is investigated (Fig 1). Recognizing secondary structures from the primary aids in the assessment of microfossils preservation examination.

#### Characterization of the primary surface in SRS resolves the shell’s textural ornamentation

Most of these structural properties are routinely investigated by SEM as optical micrographs often lack the required resolution. As shown in the previous example, SRS microscopy yields more detailed images of the microfossils thus resolving more structural details of the microfossils surface morphology. Additionally SRS tomography allows probing the internal shell architecture including spatial arrangement of chambers, their numbers, shape, subdivisions and their connections (Fig 4 and supplementary videos) and it delivers thickness information of the probed microfossil’s shell. These microfossil properties cannot be assessed by SEM on intact samples. Fig 4, C5 shows changes on the foraminiferal test wall thickness which were neither detectable by light microscopy nor by SEM (Fig 4, A5, B5 and D5). These multi-lamellar structures and possible growth rings of foraminifera test walls in this microfossil are enlarged in Fig 5, A1 and can be visualized with submicron resolution. The growth rings in Fig 5, A1 show an irregular intervallic space between 2–10 μm wide. Crucial information on the preservation state of foraminiferal tests is provided by the pores (here 0.5–3 μm in diameter) that are scattered across the surface of the test (primary structure). Typically, these pores are visible on almost all specimens except for those most heavily altered. When the pore walls are well defined with a micro-granular Mg calcite texture visible in SEM or SRS and the test wall is smooth, the sample is assumed to be well preserved (Fig 5A, 5B and 5C and supplementary information Figure A and B in S3 Fig). Otherwise, when the surface is rough and pores are not visible or filled, the sample is considered to be altered (Fig 5C and 5D). Alterations can be due to calcite infilling, neomorphism of the biogenic calcite to inorganic calcite or secondary calcite overgrowth (see next section and supplementary information illustrated in Figure E, F and I in S3 Fig).

Other primary features such as pustules and spines are resolved in SEM as well as in SRS (oriented pustules of *Heterohelix* in Fig 5E, and spines as sharper features close to the aperture in Fig 5F and Figure D in S3 Fig).

While detailed structural assessment of the microfossil surface is possible via SEM, they solely show the outer surface morphology of samples. To gain insight into the shell thickness and the internal structure in SEM the sample has to be tilted and/or broken while these measurements can be directly performed via SRS on the intact fossils with minimal sample preparation and time consumption.

#### Secondary surface structures: SRS detects secondary mineral overgrowth, mechanical abrasion and dissolution

We first elaborate on the capability of SRS to detect secondary mineral overgrowth of fossil benthic foraminifera. The surface of the specimen of the benthic foraminiferal species *Nuttallides truempyi* studied here (Fig 4, row 4 and Fig 5F) is granular and rough in both SRS and SEM images. This implies that pore walls exhibit a loose structure and developed a coarser texture altered by neomorphism showing that the microfossils are poorly preserved. These features are not resolved by conventional light microscopy hence potentially leading to a major error when interpreting stable isotope measurements.

The preservation within specimens is not always uniform. In Fig 4 D3, test details and surface structure of *Nuttallides truempyi* seem well preserved in the SEM images, but the SRS image (Fig 4 C3) reveals a rough surface, a clear indicator of calcite crystal overgrowth inside the inner wall (enlarged in Fig 5G). SRS microtomography offers advantages for detecting the poor preservation state of these specimens and contains more information on foraminiferal test preservation than SEM.

Mechanical abrasion indications such as scratches, peeling and breakage, were observed in Fig 4, D2 & D3, Fig 5, D2 & G2 and Figure B in S3 Fig. Possible reasons are transportation or sample preparation problems. Dissolution marks along microfractures (Fig 5, H enlarged from Fig 4, C3 center) and along pores (Figure C in S3 Fig) are found both with SRS and SEM imaging. The dissolution is manifested by slight enlargement and relief degradation of pores and microfractures.

SRS tomography hence promptly provides a detailed view of the entire specimen and information about surface morphology, test wall thickness, internal structure and sediment infill. Based on our SRS analyses we can ascertain that the specimens in Fig 1, samples 1 to 4 in Fig 4 and samples D-H in Fig 5 and the sample of Fig 6 are poorly preserved due to secondary calcite crystals overgrowth, cement infill and contamination, despite initial anticipations that they might be suitable for stable isotopic and trace elemental analyses.

### SRS microscopic imaging of microfossils for volumetric calculations

SRS measurements can be performed at different depths inside the microfossil with submicron depth resolution. Such compositional tomography can be used for volumetric calculations yielding information about the degree of cementation and alteration. In the following paragraph, we exemplarily conduct volumetric calculations on porosity and mineral distribution. Note that overall porosity is presented including shell pores, vacant chambers, fracture and dissolution related pores.

In stereo microscopy the sample looks well preserved, pores on the surface of the shells are visible (Fig 6A) and only some granular pyrite infillings are visible in transmitted optical microscopy (Fig 6B). Also, SEM shows a smooth surface with pores and no apparent textural alterations (Fig 6C). However, by using SRS tomography intense calcite cementation inside the fossil is revealed (Fig 6D). This calcite cementation renders the specimen unsuitable for stable isotopic analysis.

To illustrate porosity and mineralogy distribution in the studied samples a middle slice, 35 μm below the shell surface is displayed (Fig 6D and 6E). Fig 6D shows the Mg calcite distribution (turquoise, vibrational resonance at 1092 cm^-1^) and light-absorbing species iron oxides and/or pyrite (red, broad spectral response 1080–1140 cm^-1^), 35 μm below the shell surface. Fig 6E represents a segmented image calculated from D indicating porosity distribution (black).

Volumetric calculations with MATLAB revealed 37% of the scanned sample is porous (including shell porosity and vacant chambers), 10% contains iron oxides and pyrite and the remaining 53% is Mg calcite. However, distinguishing primary Mg calcite as the building material of fossil shell and secondary Mg calcite as cement infill is not straightforward but could in principle be scrutinized by a crystallinity analysis. Here, looking at the mineral’s response to different polarizations of the incoming excitation light could yield information about the crystal structure and orientation but this is beyond the scope of this work.

The SRS maximum projection in Fig 6F is mapping surface details of the foraminifer and reveals similar information as deduced from the SEM image. This can also be used for shell surface porosity calculation and pore shape and distribution density calculations. In this sample 23% of the shell surface is porosity. This includes tests primary pores and mechanical abrasion. Using this measure the number of pores can be calculated from the average pore diameter.

## Conclusions and perspectives

In this study, we successfully employed stimulated Raman microspectroscopy and tomography to investigate the 3D morphology and composition of fossil foraminifera, with the goal of resolving post-depositional alteration. Via side-by-side comparison with the current state-of-the-art approaches–optical microscopy and SEM–we demonstrate that this technique yields more information about the fossil uncovering alterations that cannot be detected with conventional methods. These results on relevant microfossils convincingly show that SRS microtomography in combination with pump-probe spectroscopy can become a powerful tool in geological research enabling the identification of complex microfossil species and selecting specimens suitable for geochemical analyses, for example, for stable oxygen isotope and trace elemental analyses.

The information obtained using SRS includes tomographical information such as chamber arrangement, wall porosity distribution, test wall thickness, chambers infill, and the presence of overgrowths and/or neomorphic mineral phases on the micrometric scale. While exterior preservation of the microfossil can be assessed using a SEM (dissolution, neomorphism and authigenic mineral overgrowth) and to a lesser extend also by optical microscopy, imaging the interior structure, however, is difficult to perform using SEM alone. Here, SRS has a clear advantage over commonly used methods as it is able to visualize and analyze fossils internally with high spatial resolution in a non-destructive and time efficient manner.

The position and shape of spectral bands allow for *in situ* identification of a wide range of minerals (including different polymorphs) and compounds by SRS and may indicate the presence of secondary minerals in the fossil or replacement of the fossil test. However, if replacement happens with the same mineral without a change in morphology, texture, and polymorphs, the identification with SRS would be challenging and other methods will be required to reveal such processes.

Our results confirm that SRS can be effectively applied to assess the degree of preservation of microfossil specimens, for deducing their pristine composition or to explain the acquired geochemical data in terms of alterations. In cases documented in this study, the specimens looked adequate for chemical analysis as confirmed by optical and electron microscope visualization. However, some critical alterations could only be detected by means of SRS analyses at the micrometric scale.

The SRS technique is applicable to specimens up to a few hundred microns in size allowing to infer the mineralogy and porosity distribution of the sample in 3D without applying any treatments. Furthermore, the technique is scalable in a fashion similar to flow cytometry. A current drawback is the complexity and price of a SRS microscope. This could, however, be simplified dramatically at much lower costs by new fiber laser design.

Overall, we have demonstrated a powerful method to efficiently assess the preservation state of microfossils. The demonstrated methodology may help to greatly improve the quality of geochemical data and their interpretation, which will have great implications in the field of paleoclimatology.

## Supporting information

S1 Fig Principle of SRS microscopy. A: In stimulated Raman loss, the Stokes pulse train (red) is modulated. Upon pulse overlay and Raman transitions, the unmodulated Pump signal (blue) experiences a modulation transfer *I*_*pump*_*—I*_*mod*_. B-C: Technical implementation of SRS microscopy. B: Sample configuration for SRS imaging. The excitation light passes an objective and is collected by a condenser (lens) before the pump light is detected by a photodiode. C: Principle of SRS detection using a lock-in amplifier.
(TIF)

S2 Fig Raman spectra of two different microfossils. A clear peak at 1092cm^-1^ on top of an auto-fluorescence background is visible indicating the presence of calcite in both samples.
(TIF)

S3 Fig SEM images of different wall surfaces showing post-depositional alteration as well as well-preserved surfaces. (A) Well preserved test wall texture with pores. (B) Scratches and mechanical removal of outer wall. (C) Dissolution features around pores. (D) Spines on the shell surface of *Heterohelix*. (E) Neomorphism and cementation on outer test surface. (F) Neomorphism (recrystallization) of badly preserved shell. (G) Contamination of pores with coccolith and presence of small calcite overgrowth. (H) Contamination by authigenic minerals on surface of *Heterohelix*. (I) Pervasive calcite infilling of pores.
(TIF)

S1 Video 360 deg maximum projection of benthic foraminiferal species *Nuttallides*. *truempyi*. (AVI)

S2 Video 360 deg maximum projection of benthic foraminiferal species *Nuttallides*. *truempyi*. (AVI)

S3 Video 360 deg maximum projection of planctonic foraminiferal genus *Heterohelix*. (AVI)
