# Supplementary figures and images for: Improving preservation state assessment of carbonate microfossils in paleontological research using label-free stimulated Raman imaging
Source: PLoS One. 2018 Jul 11;13(7):e0199695. doi: 10.1371/journal.pone.0199695 (PMC6040746; doi:10.1371/journal.pone.0199695)

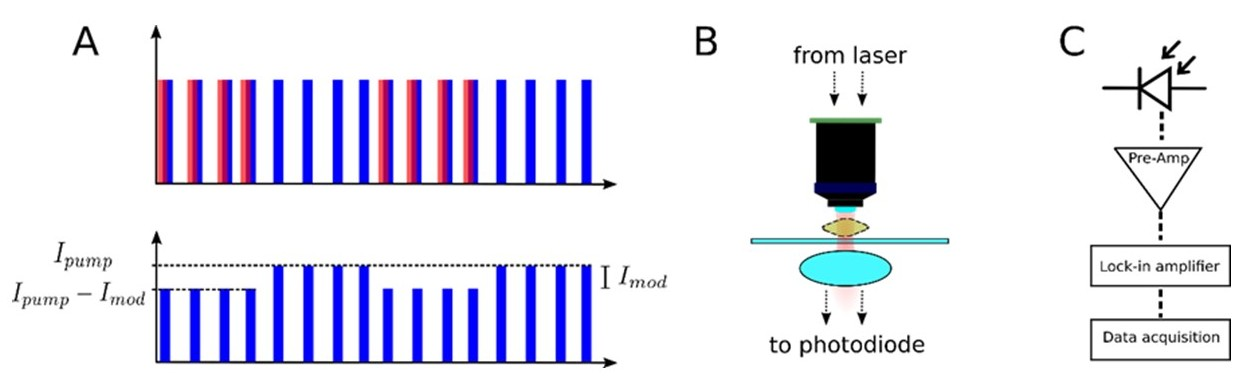

Supplement: S1 Fig — A: In stimulated Raman loss, the Stokes pulse train (red) is modulated. Upon pulse overlay and Raman transitions, the unmodulated Pump signal (blue) experiences a modulation transfer Ipump—Imod. B-C: Technical implementation of SRS microscopy. B: Sample configuration for SRS imaging. The excitation light passes an objective and is collected by a condenser (lens) before the pump light is detected by a photodiode. C: Principle of SRS detection using a lock-in amplifier. (TIF) [file pone.0199695.s001.tif]

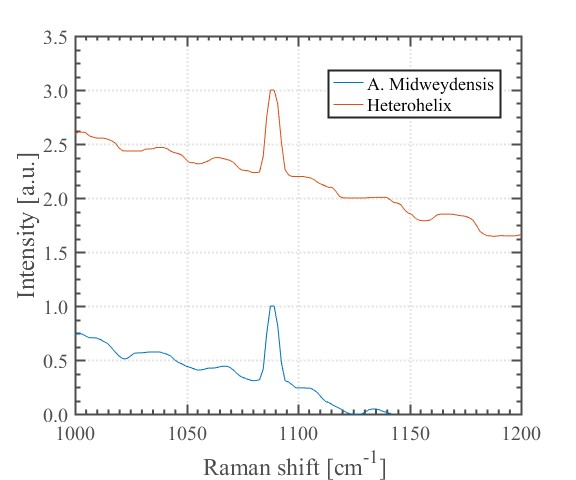

Supplement: S2 Fig — A clear peak at 1092cm-1 on top of an auto-fluorescence background is visible indicating the presence of calcite in both samples. (TIF) [file pone.0199695.s002.tif]

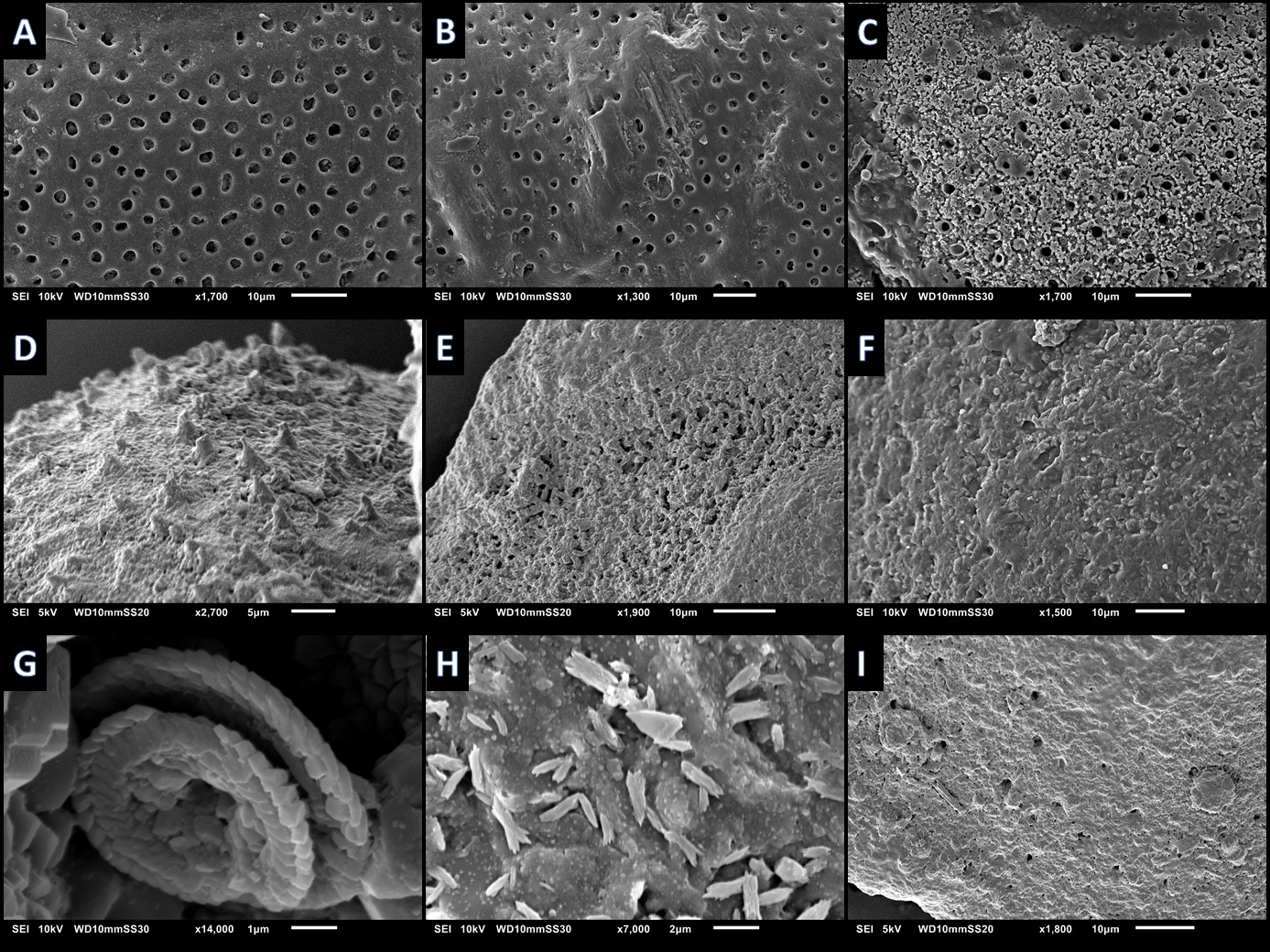

Supplement: S3 Fig — (A) Well preserved test wall texture with pores. (B) Scratches and mechanical removal of outer wall. (C) Dissolution features around pores. (D) Spines on the shell surface of Heterohelix. (E) Neomorphism and cementation on outer test surface. (F) Neomorphism (recrystallization) of badly preserved shell. (G) Contamination of pores with coccolith and presence of small calcite overgrowth. (H) Contamination by authigenic minerals on surface of Heterohelix. (I) Pervasive calcite infilling of pores. (TIF) [file pone.0199695.s003.tif]
